# Predisposition to Alzheimer’s and Age-Related Brain Pathologies by PM2.5 Exposure: Perspective on the Roles of Oxidative Stress and TRPM2 Channel

**DOI:** 10.3389/fphys.2020.00155

**Published:** 2020-02-26

**Authors:** Lu Wang, Lin Yu Wei, Ran Ding, Yanyan Feng, Dongliang Li, Chaokun Li, Philippa Malko, Sharifah A. Syed Mortadza, Weidong Wu, Yaling Yin, Lin-Hua Jiang

**Affiliations:** ^1^Sino-UK Joint Laboratory of Brain Function and Injury and Department of Physiology and Neurobiology, Xinxiang Medical University, Xinxiang, China; ^2^School of Biomedical Sciences, Faculty of Biological Sciences, University of Leeds, Leeds, United Kingdom; ^3^Department of Physiology, Sanquan College of Xinxiang Medical University, Xinxiang, China; ^4^Department of Biochemistry, Faculty of Biotechnology and Biomolecular Sciences, Universiti Putra Malaysia, Seri Kembangan, Malaysia; ^5^School of Public Heath, Xinxiang Medical University, Xinxiang, China

**Keywords:** Alzheimer’s disease, age-related brain pathologies, PM2.5, oxidative stress, TRPM2 channel

## Abstract

Accumulating epidemiological evidence supports that chronic exposure to ambient fine particular matters of <2.5 μm (PM2.5) predisposes both children and adults to Alzheimer’s disease (AD) and age-related brain damage leading to dementia. There is also experimental evidence to show that PM2.5 exposure results in early onset of AD-related pathologies in transgenic AD mice and development of AD-related and age-related brain pathologies in healthy rodents. Studies have also documented that PM2.5 exposure causes AD-linked molecular and cellular alterations, such as mitochondrial dysfunction, synaptic deficits, impaired neurite growth, neuronal cell death, glial cell activation, neuroinflammation, and neurovascular dysfunction, in addition to elevated levels of amyloid β (Aβ) and tau phosphorylation. Oxidative stress and the oxidative stress-sensitive TRPM2 channel play important roles in mediating multiple molecular and cellular alterations that underpin AD-related cognitive dysfunction. Documented evidence suggests critical engagement of oxidative stress and TRPM2 channel activation in various PM2.5-induced cellular effects. Here we discuss recent studies that favor causative relationships of PM2.5 exposure to increased AD prevalence and AD- and age-related pathologies, and raise the perspective on the roles of oxidative stress and the TRPM2 channel in mediating PM2.5-induced predisposition to AD and age-related brain damage.

## Introduction

Air pollution has increasingly become an environmental risk to public health worldwide, particularly to people living in large cities. This has been supported by compelling evidence for strong association of chronic exposure to ambient air pollution with increased morbidity and mortality of respiratory and cardiovascular diseases (Liu et al., 2019). There is growing evidence to show that exposure to polluted ambient air is also injurious to the brain (Brockmeyer and D’Angiulli, 2016; Clifford et al., 2016; Power et al., 2016; Babadjouni et al., 2017; Cohen et al., 2017; Sripada, 2017; Underwood, 2017; Bencsik et al., 2018). Among other air pollutants, the fine particulate matters (PM) with an aerodynamic diameter of <2.5 μm (PM2.5), which include ultrafine PM with a size of <200 nm (PM0.2) and nanometer-sized PM (nPM) or nanoparticles (NPs), has attracted particular attentions for their potential damage to the brain because they more readily enter the brain; they can penetrate the olfactory epithelium and, alternatively and/or additionally, travel deep into the airways and lungs, infiltrate into the blood circulation, and finally cross the blood–brain barrier (BBB) (Heusinkveld et al., 2016; Maher et al., 2016; Underwood, 2017; Bencsik et al., 2018). Such tiny particles in ambient air can be mainly derived from diesel exhaust (DE) and traffic/combustion-related air pollution, and also increasingly result from manufacturing, application, and subsequent release of nanomaterials (Bencsik et al., 2018). In general, the smaller their size, the greater their capacity of inducing oxidative stress and thus the more severe the resulting cytotoxicity is (Underwood, 2017). It has been shown that exposure to traffic-related air pollution and PM2.5 during early life can damage brain and cognitive development and increase the prevalence of autism spectrum disorders (Raz et al., 2015; Saenen et al., 2015; Sunyer et al., 2015; Sram et al., 2017; Sripada, 2017; Fu et al., 2019; Jo et al., 2019; Pagalan et al., 2019a, b). Accumulating evidence also supports predisposition by PM2.5 exposure of both children and adults to various brain pathologies including Alzheimer’s disease (AD), Parkinson’s disease, amyotrophic lateral sclerosis, and stroke and depressive disorders (Mushtaq et al., 2015; Kioumourtzoglou et al., 2017; Seelen et al., 2017; Sram et al., 2017; Bencsik et al., 2018; Bazyar et al., 2019; Fu et al., 2019; Shou et al., 2019). Air pollution has increasingly become a major environmental risk factor inducing dementia (Underwood, 2017). AD represents the most common cause of age-related brain damage and dementia. In this article, we discuss the studies showing predisposition by PM2.5 exposure to AD and age-related brain damage, and hypothesize the roles of oxidative stress and the oxidative stress-sensitive transient receptor potential melastatin 2 (TRPM2) channel in PM2.5-induced AD and age-related brain pathologies.

## Alzheimer’s Pathologies and Oxidative Stress

Alzheimer’s disease is an age-related neurodegenerative condition manifested by progressive decline and loss of cognitive function. AD patients in the later disease stage suffer brain structural alterations, including shrinking of hippocampus and cerebral cortex (Drew, 2018). Prior to such structural atrophy, the AD brain is more often than not characterized at the microscopic level by widespread aggregation and deposition of extracellular amyloid β (Aβ) peptides in amyloid plaques and intra-neuronal hyper-phosphorylated tau proteins into neurofibrillary tangles (NFT). Genetically, AD can be familial and sporadic. Familiar AD (FAD), identified in a very small number of cases, arises predominantly from mutations in amyloid precursor protein (APP), presenilin 1 (PS1), and PS2 that lead to excessive Aβ generation and neurotoxic Aβ fibrillary formation, a process often referred to as amyloidogenesis. Sporadic form, accounting for a vast majority of cases, results from aging, genetic [e.g. carrying apolipoprotein E (APOE) ε4 allele which is associated with a reduced capacity of clearing and degrading Aβ], and environmental risk factors that aggravate amyloidogenesis (Blennow et al., 2010; Buxbaum, 2019; Licher et al., 2019).

The amyloid cascade hypothesis of AD posits that Aβ directly or indirectly causes synaptic deficits and neuronal loss, leading to cognitive dysfunction (Blennow et al., 2010; Selkoe and Hardy, 2016). The direct toxicity of Aβ to synapses and neurons is well attested by *in vitro* studies exposing cultured neurons to Aβ alongside *in vivo* studies using various strains of transgenic AD mice that express AD-linked human mutant genes leading to elevated Aβ levels (e.g. APP/PS1, 5xFAD, or APOE ε4 mice) or wild-type animals, predominantly mice and rats, with intra-hippocampal administration of neurotoxic Aβ (Blennow et al., 2010; Buxbaum, 2019). Recent studies have disclosed AD-related alterations in the genetic and functional profile of microglia, the immune-competent cells in the brain, and association of mutations in microglia-specific genes (e.g. TREM2) with AD, which triggers an escalating interest in microglia, particularly microglia-mediated neuroinflammation, and recognition of its importance in AD pathogenesis and progression (McQuade and Blurton-Jones, 2019; Wang and Colonna, 2019). The brain is highly vulnerable to oxidative damage, due to its high oxygen consumption, high content of fatty acids, and weak antioxidant capacity. Aβ can promote ROS generation and in return ROS can enhance Aβ generation and aggregation. ROS are potent in modifying functionally important molecules (e.g. DNA and proteins) and damaging intracellular organelles (e.g. lysosomes and mitochondria) (Jiang et al., 2016; Butterfield, 2018). Aβ and ROS synergistically can damage synapses and neurons, induce microglial activation and neuroinflammation, and impair neurovascular and BBB function (Sweeney et al., 2018). Oxidative damage is a prominent and common feature of many neurodegenerative diseases and accepted as an important neurodegeneration mechanism (Jiang et al., 2016; Butterfield, 2018; Trist et al., 2019).

## Causative Relationship Of PM2.5 Exposure With AD And Age-Related Pathologies

### PM2.5 Exposure Induces Predisposition to Dementia, AD, and Age-Related Brain Damage

**FIGURE 1:**
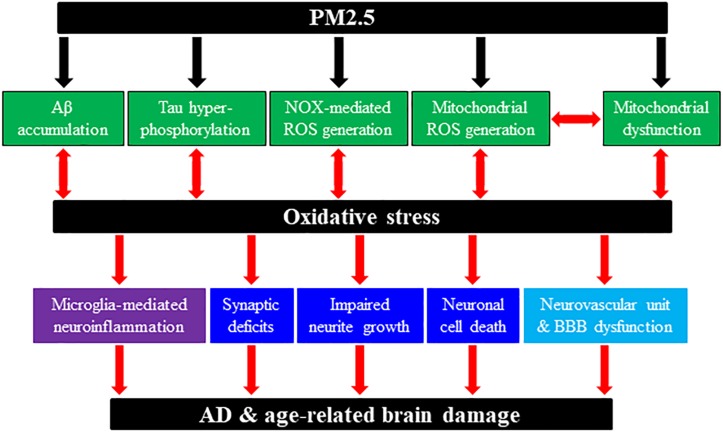
Schematic summary of potential molecular and cellular pathways involved in PM2.5-induced predisposition to AD and age-related brain damage. Chronic exposure to PM2.5 in ambient polluted air can predispose humans to AD or rodents to AD-related brain damage through generation or activation of multiple pathological factors and pathways. PM2.5 exposure can induce or enhance amyloid β (Aβ) accumulation; tau hyper-phosphorylation; NADPH oxidase (NOX)-mediated reactive oxygen species (ROS) generation; mitochondrial dysfunction; and mitochondrial ROS generation. In addition, PM2.5 exposure-induced AD-related brain pathologies engage multiple cellular pathways, including synaptic deficits, impaired neurite growth and cell death in neurons; microglia (and astrocytes) activation and generation of neurotoxic proinflammatory meditators [e.g., interleukin (IL)-1β, tumor necrosis factor-α, IL-6, ROS]; neurovascular unit and blood–brain barrier (BBB) dysfunction. We have hypothesized the roles of oxidative stress and subsequent activation of the TRPM2 channel (not depicted here) in coupling PM2.5 exposure to predisposition to AD and age-related brain damage leading to cognitive dysfunction.

The interest in PM2.5-induced brain damage and cognitive dysfunction was in fact triggered by a histochemical study revealing widespread pathological modifications (e.g. degenerating cortical neurons, apoptotic white matter glial cells, NFT, and BBB impairment) in the brains of demented dogs living in a highly air polluted urban region of Mexico City (Calderon-Garciduenas et al., 2002). Subsequent studies by the same group have documented numerous early pathological indicators of neurodegenerative diseases, including accumulation of Aβ42, oxidative stress, neuroinflammation, and neurovascular damage in the brains of children and young people in Mexico City experiencing chronic exposure to high levels of air pollution and PM2.5 (Calderon-Garciduenas et al., 2004, 2008, 2012, 2016, 2018; Gonzalez-Maciel et al., 2017). Consistently, epidemiological studies support significant association of chronic exposure to PM2.5 or traffic-related air pollution with increased risk to dementia and AD (Kioumourtzoglou et al., 2016; Chen et al., 2017; Fu et al., 2019) and age-related cerebral atrophy (Wilker et al., 2015). Chronic PM2.5 exposure has also been associated with accelerated loss of gray and white matters or increased risk of cognitive impairment in older women (Casanova et al., 2016; Cacciottolo et al., 2017). Collectively, these studies support predisposition to AD and age-related brain damage by chronic PM2.5 exposure. Further supporting evidence comes from studies examining cognitive function and neuro-behaviors in rodents after exposure to ambient PM2.5 with various doses and durations. An early study using 4-week-old male mice reported that PM2.5 exposure (94.4 μg/m^3^, 6 h per day, and 5 days per week) for 10 months impaired learning and memory and also resulted in depressive-like responses (Fonken et al., 2011). A recent study shows that PM2.5 exposure (3 mg/kg every other day) for 4 weeks also damaged learning and memory in young mice (4 weeks). However, such PM2.5 exposure-induced detrimental effects were not observed in adult (4 months) and middle-aged (10 months) mice (Ning et al., 2018), suggesting an age-ceiling effect. Another recent study using 2-month-old male rats reports that intra-tracheal injection of PM2.5 (20 mg/kg every 7 days) for 3–12 months damaged sensory functions as well as learning and memory (Zhang et al., 2018). As discussed in detail below, studies provide further evidence to suggest that PM2.5 exposure predisposes humans to AD and development of AD-related pathologies in rodents via exacerbating the pathological pathways that are known to be implicated in AD, namely, directly causing synaptic deficits and neuronal cell death, or indirectly inducing microglia-mediated neuroinflammation and disrupting neurovascular function (Figure 1).

### PM2.5 Exposure Impairs Neurite Growth, Expression of Synapse Proteins and Receptors, and Neuronal Cell Viability

Studies have investigated the effects of PM2.5 exposure on neurons, drawing particular attention to neurite growth, synaptic structure and function, and neuronal cell viability. In the above-mentioned study, exposure of 4-week-old mice to PM2.5 for 10 months reduced dendritic spine density of hippocampal neurons in the CA1 region and also dendritic length and branching in the CA3 region (Fonken et al., 2011). Another study using 8-week-old male mice showed that exposure to PM2.5 (65.7 ± 34.2 μg/m^3^, 6 h per day, and 5 days per week) for 9 months induced synaptic alterations by increasing the expression of postsynaptic density protein 95 (PSD95) without effect on the expression of presynaptic protein synaptophysin (Bhatt et al., 2015). A recent study using 3-month-old mice shows that exposure to ambient nPM (5 h per day and 3 days per week) for 10 weeks caused white matter damage in the CA1 and DG regions of the hippocampus, and suppressed neurite outgrowth in the CA1 region (Woodward et al., 2017). The same study examined the receptors for glutamate, the key excitatory neurotransmitter in the hippocampus. The expression of α-amino-3-hydroxy-5-methyl-4-isoxazolepropionic acid (AMPA) class receptor GluR1 subunit was reduced, whereas the expression of GluR2 subunit, or *N*-methyl-D-aspartate (NMDA) class receptor NR2A or NR2B subunit remained unchanged. Such nPM-induced effects on white matter and GluR1 expression in 3-month-old mice were similar to these in 18-month-old mice due to aging. Moreover, in the old mice, nPM exposure induced no further detrimental effect, again indicating an age-ceiling effect (Woodward et al., 2017). Another recent study shows that exposure of female mice to nPM for 10 weeks selectively reduced neurite density in the CA1 region and attenuated the GluR1 expression without effect on the expression of GluR2, NR2A, NR2B, synaptophysin, and PSD95 (Cacciottolo et al., 2017). The study also shows that nPM exposure selectively decreased neurite density and the GluR1 expression in hippocampus of 5xFAD^+/–^ mice carrying the human APOE ε4 gene as observed in wild-type mice. In addition, PM2.5-induced AD-related pathologies are associated with neuronal death. For example, a recent study demonstrates that intra-tracheal injection of PM2.5 in 2-month-old male rats induced necrosis and apoptosis of cortical neurons (Zhang et al., 2018). A more recent study using human neuroblastoma SH-SY5Y cells, a widely used cell model in the study of neurodegeneration mechanism (Xicoy et al., 2017), also shows that exposure to PM2.5 (25–250 mg/mL) for 24 h reduced cell viability (Wang et al., 2019). Collectively, these studies suggest that PM2.5 exposure can lead to neurodegeneration by compromising neurite growth, expression of synapse proteins and receptors, and neuronal cell viability (Figure 1).

### PM2.5 Exposure Induces Microglial Cell Activation and Generation of Proinflammatory Cytokines

As has been introduced above, microglia-mediated neuroinflammation has attracted increasing attention for its role in AD. Interleukin (IL)-1β, tumor necrosis factor (TNF)-α, and IL-6 are the major neurotoxic pro-inflammatory cytokines in AD-related neuroinflammation. Studies, both *in vivo* using rodents and *in vitro* using cultured cells, have provided evidence to suggest that PM2.5 exposure can induce deleterious effects on the brain via neuroinflammation, mainly through excessive generation of these proinflammatory cytokines by microglia (Brockmeyer and D’Angiulli, 2016; Jayaraj et al., 2017). An early study showed that exposure of 4-week-old male mice to PM2.5 for 10 months upregulated the expression of IL-1β and TNF-α in the brain (Fonken et al., 2011). Another early study using 12-to-14-week-old male rats reported that exposure to DE (0.5 and 2.0 mg/m^3^, 4 h per day, and 5 days per week) for 1 month resulted in concentration-dependent increases in the expression of ionized calcium-binding adaptor molecule 1 (Iba-1), a microglial cell marker, and IL-6 in the whole brain (Levesque et al., 2011). More specifically, such exposure elevated the levels of Iba-1, IL-1β, IL-6, and TNF-α in the cortex and midbrain regions. The same study also showed that intra-tracheal administration of single high dose of DE-derived particles (20 mg/kg) increased the TNF-α level in the serum and whole brain and that exposure to DE-derived nPM (50 μg/mL) enhanced TNF-α generation from rat immortalized microglial cells prior primed by lipopolysaccharide (LPS) (Levesque et al., 2011). Similarly, a recent study using 3-month-old mice shows that exposure to ambient nPM for 10 weeks increased the Iba-1 expression in the CA1 and DG regions of the hippocampus and the TNF-α expression level in the whole brain (Woodward et al., 2017). Another recent study using 3-month-old mice also reports that exposure to DE containing 250–300 μg/m^3^ PM2.5 for 6 h induced morphological changes of microglial cells and elevated Iba-1 expression in the hippocampus, similarly in male and female mice (Cole et al., 2016). Furthermore, nPM exposure massively elevated the levels of IL-1β, IL-6, TNF-α, and IL-3 and the level of malondialdehyde (MDA), a biomarker of oxidative stress, in the hippocampus, and such brain inflammation and oxidative stress were noticeably higher in male mice than in female mice, suggesting sex difference (Cole et al., 2016). A separate study using cultured rat microglial and astrocytes demonstrated that exposure to traffic-derived PM0.2 (6–12 μg/ml) induced the TNF-α expression (Cheng et al., 2016). The study also showed that the neurite length of rat cortical neurons, when cultured in media conditioned by PM0.2-exposed microglia, astrocytes, or mixed cell cultures, was significantly shorter. PM0.2-induced reduction in neurite growth was prevented by siRNA-mediated knockdown of the TNF-α expression, indicating that PM0.2-induced TNF-α generation by glial cells mediates such neurotoxicity (Cheng et al., 2016). Another recent study using 15/16-week-old mice shows that exposure to traffic-derived nPM (330 μg/m^3^, 5 h per day, and 3 days per week) for 10 weeks induced microglial cell activation and increased the deposition of complement C5/C5α proteins and C5a receptor 1 in the corpus callosum (Babadjouni et al., 2018). Taken together, these studies support that PM2.5 exposure causes AD- and age-related brain pathologies via inducing neuroinflammation (Figure 1).

Both *in vivo* studies using APP/PS1 mice and *in vitro* studies using cultured microglial cells have revealed an important role for Aβ-induced activation of the multi-protein complex NLRP3 inflammasome and caspase-1 and ensuring generation of IL-1β in AD pathologies (Heneka et al., 2015; Wes et al., 2016; White et al., 2017). Consistently, a recent study using LPS-primed cultured mouse microglial cells shows that exposure to PM2.5 (50 μg/ml) for 4 h enhanced Aβ-induced NLRP3 inflammasome activation and IL-1β generation (Wang et al., 2018). Such PM2.5-induced effects were dependent upon both NADPH oxidase (NOX)- and mitochondria-dependent generation of ROS. Furthermore, PM2.5 exposure enhanced the capacity of Aβ-treated microglia to induce neuronal cell death in cortical neuron/microglia co-cultures, where microglia and neurons were separately seeded in the upper and lower chambers, respectively, in trans-well plates. Such PM2.5-induced microglia-mediated neuronal cell death was prevented by pharmacological inhibition of NOX-mediated and mitochondrial ROS generation or caspase-1 activation (Wang et al., 2018). These results therefore suggest that PM2.5 exposure can induce neuroinflammation via sequential induction of oxidative stress, NLRP3 inflammasome activation, and IL-1β generation.

### PM2.5 Exposure Induces Neurovascular Unit and BBB Dysfunction

Impairment in the neurovascular unit and BBB function can render enhanced infiltration of peripheral immune cells and proinflammatory mediators to intensify brain inflammation as well as the entry of PM2.5 into the brain. Evidence exists to indicate that Aβ can induce neurovascular unit and BBB dysfunction and thereby increase susceptibility to AD (Park et al., 2014; Sweeney et al., 2018). As mentioned above, histochemical studies of the brain of young people who lived in Mexico City suggest that chronic exposure to PM2.5 or combustion-derived nNPs can compromise the neurovascular unit and BBB function (Calderon-Garciduenas et al., 2016; Gonzalez-Maciel et al., 2017) and thereby aggravate AD-related pathologies (Figure 1).

## Induction of Ad-Associated Molecular Alterations by Pm2.5 Exposure

### PM2.5 Exposure Enhances Aβ Accumulation and Tau Hyper-Phosphorylation

There is evidence to indicate that PM2.5 exposure enhances the levels of Aβ and tau hyper-phosphorylation. As shown in an aforementioned recent study using 8-week-old male mice, exposure to ambient PM2.5 (65.7 ± 34.2 μg/m^3^, 6 h per day, and 5 days per week) for 9 months reduced the level of APP protein and increased the levels of beta-site APP cleaving enzyme (BACE) protein and Aβ40 in the cerebral temporal cortex (Bhatt et al., 2015). A recent study using 2-month-old female 5xFAD^+/–^/APOE ε4 mice reports that nPM exposure for 15 weeks accelerated amyloid deposition and plaque formation and elevated the level of Aβ oligomers, which may contribute to nPM-induced selective reduction in neurite density in the CA1 region (Cacciottolo et al., 2017). The same study also shows that exposure of mouse neuroblastoma N2a cells expressing Swedish mutant APP to 10 μg/ml nPM for 24 h enhanced Aβ42 generation. Another recent study using 10-week-old female 5xFAD mice reports that exposure to DE (0.95 mg/m^3^, 6 h per day, and 5 days per week) for 3 weeks elevated the levels of cortical Aβ plaque load and whole brain Aβ42 (Hullmann et al., 2017). However, prolonged exposure for 13 weeks resulted in no effect on the levels of Aβ plaque load and whole brain Aβ42, which were already high due to aging and AD progression (Hullmann et al., 2017). Such an observation further indicates an age-related ceiling effect as previously reported in wild-type mice (Woodward et al., 2017). Another recent study using 10-month-old mice has found that exposure to ambient PM2.5 (3 mg/kg) for 4 weeks increased the level of tau hyper-phosphorylation as well as altering neuronal mitochondria, inducing ROS generation and reducing the cellular ATP content (Gao et al., 2017). Therefore, PM2.5 exposure can induce AD-related pathologies via stimulating Aβ generation and tau hyper-phosphorylation (Figure 1).

### PM2.5 Exposure Stimulates ROS Generation and Oxidative Stress

As discussed above, excessive ROS generation and ensuing oxidative damage play an important role in AD. There is increasing evidence to indicate that PM2.5-induced AD-related pathologies are associated with increased ROS generation and oxidative stress. For example, a recent study using 2-month-old male rats shows that intra-tracheal injection of PM2.5 significantly reduced the activities of superoxide dismutase (SOD), a superoxide radical scavenger, and glutathione (GSH) peroxidase, an important antioxidant enzyme catalyzing the reduction of hydrogen peroxide (H_2_O_2_) by GSH, and increased the level of MDA (Zhang et al., 2018). There were also substantial mitochondrial dysfunction and loss of cristae within mitochondria in cortical neurons of PM2.5-exposed rats (Zhang et al., 2018). A more recent study using SH-SY5Y cells shows that exposure to PM2.5 (25, 100, and 250 mg/mL) concentration-dependently increased the levels of intracellular Ca^2+^ and ROS, and reduced the cellular ATP content and GSH/GSSG ratio (Wang et al., 2019). PM2.5 exposure also induced mitochondrial fragmentation and increased the level of optic atrophy 1 (OPA1) protein, which is critical for mitochondrial fusion, without change in the level of dynamin-related protein 1 (Drp1) protein, which is important for mitochondrial fission. Furthermore, PM2.5 exposure triggered the opening of mitochondrial permeability transition pore, decreased the mitochondrial membrane potential and mitochondrial SOD activity, and elevated the mitochondrial content of MDA (Wang et al., 2019). Therefore, accumulating evidence suggests that ambient PM2.5 exposure induces oxidative stress and mitochondrial dysfunction, leading to neuronal cell death (Figure 1).

## Perspective on Roles of Oxidative Stress and Trpm2 Channel in Pm2.5-Induced Disposition to Ad and Age-Related Brain Damage

### TRPM2 Channel Is Critical in Mediating AD- and Age-Related Cognitive Dysfunction

**FIGURE 2:**
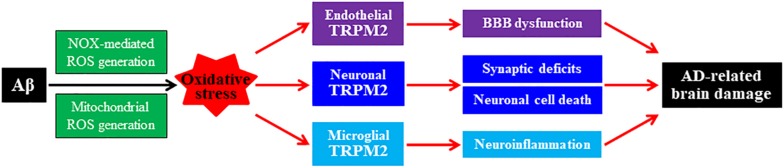
Roles of oxidative stress and TRPM2 channel in mediating Aβ-induced AD-related brain damage. Chronic exposure to elevated levels of amyloid β peptide (Aβ) induces NADPH oxidase (NOX)-mediated and mitochondrial generation of reactive oxygen species (ROS), giving rise to oxidative stress in neuronal, microglial, and endothelial cells in the brain. Activation of the TRPM2 channels in these cells by ROS or under oxidative stress, respectively, mediates synaptic deficits, neuronal cell death, microglial cell activation and generation of neurotoxic proinflammatory mediators, neurovascular unit, and blood–brain barrier (BBB) dysfunction. These changes contribute to Alzheimer’s disease (AD)-related brain damage leading to cognitive dysfunction.

The TRPM2 channel, member of the mammalian TRP channel superfamily, is a Ca^2+^-permeable cationic channel gated by intracellular ADP-ribose (ADPR) and related compounds (Perraud et al., 2001; Yu et al., 2017, 2019). The TRPM2 channel is highly sensitive to ROS due to the potent capacity of ROS to promote ADPR generation (Jiang et al., 2010). Studies using transgenic TRPM2-knockout (TRPM2-KO) mice and/or derived cell cultures provide compelling evidence to show the TRPM2 channel expression in neurons, microglia, astrocytes, neuro-endothelial cells, and pericytes in the brain and demonstrate its crucial role in mediating brain damage induced by various pathological conditions (Li et al., 2015; Jiang et al., 2018; Malko et al., 2019; Mai et al., 2020). For example, a recent study introducing TRPM2-KO in the APP/PS1 mice has disclosed a key role of the TRPM2 channel in Aβ-induced synaptic deficits, microglial cell activation, and age-related impairment in learning and memory (Ostapchenko et al., 2015). Studies using cultured hippocampal neurons have revealed TRPM2 channel activation to be essential in a positive feedback loop that couples ROS/Aβ-induced NOX-mediated and mitochondrial ROS generation, lysosomal and mitochondrial dysfunction to neuronal cell death (Li et al., 2017; Li and Jiang, 2018, 2019). Studies using cultured microglial cells support a critical role for the TRPM2 channel in microglial cell activation, ROS generation, and production of proinflammatory cytokines induced by exposure to ROS, Aβ, and other pathological stimuli (Aminzadeh et al., 2018; Syed Mortadza et al., 2018). In addition, there is evidence to indicate a significant role for the TRPM2 channel in endothelial cells in mediating Aβ-induced oxidative damage to the neurovascular unit and BBB function (Park et al., 2014). As illustrated in Figure 2, accumulating evidence supports the roles of oxidative stress and the TRPM2 channel in AD-related pathologies via mediating Aβ-induced synaptic deficits, neuronal cell death, microglia-mediated neuroinflammation, and neurovascular and BBB dysfunction (Jiang et al., 2018).

A recent study has examined the effect of TRPM2-KO on age-related loss of cognitive function in mice (Kakae et al., 2019). There was noticeable decline in working and cognitive memory in middle-age WT mice at the age of 12–16 months and significant impairment in spatial memory in aged WT mice reaching 20–24 months compared with young WT mice of 2–3 months. Such age-related cognitive dysfunction in WT mice was not observed in age-matched TRPM2-KO mice. This study also has documented substantial white matter damage, loss of neuronal cells in hippocampus, and an increase in the number of Iba1-positive microglial/macrophage cells and CD3-positive T cells and a greater level of TNF-α in the corpus callosum and hippocampus in aged WT mice. Interestingly, all of these age-related detrimental effects were almost completely prevented by TRPM2-KO. These results provide clear evidence to suggest an important role for the TRPM2 channel in mediating age-related loss of cognitive function, at least in part via neuroinflammation.

### Oxidative Stress and TRPM2 Channel Activation Are Important in NPs-Induced Damaging Effects

There is increasing evidence to show important roles for ROS generation and TRPM2 channel activation in mediating multiple cellular effects induced by ultrafine PM, particularly various types of NPs. For example, our study showed that in human embryonic kidney 293 (HEK293) cells expressing a low level of the TRPM2 channel, exposure to silica NPs for 3–6 h initially induced TRPM2-independent generation of ROS, which sufficiently activated the TRPM2 channel and up-regulated the NOX2 expression to further provoke oxidative stress and subsequent cell death (Yu et al., 2015). Intriguingly, silica NPs-induced cell death in HEK293 cells was attenuated by elevating the TRPM2 expression. This was due to selective and TRPM2-dependent down-regulation of the NOX4 expression and ROS generation. There was similar TRPM2 expression-dependence of silica NPs-induced cell death in bone marrow-derived macrophages. Collectively, this study suggests a dual role of the TRPM2 channel in NPs-induced effect on cell viability. A recent study also supports a significant role of the TRPM2 channel in mediating the cytotoxicity of mesoporous silica NPs to HEK293 cells (Mohammadpour et al., 2019). A separate study showed that exposure to lanthanide-based nanoparticles (LNs) induced NLRP3 inflammasome activation and IL-1β generation from LPS-primed mouse bone marrow-derived macrophages, human THP-1, and mouse peritoneal macrophages *in vitro* and also from mice intraperitoneally injected with LNs *in vivo* (Yao et al., 2016). LNs-induced NLRP3 inflammasome activation and IL-1β generation were reduced by inhibiting mitochondrial ROS generation and strongly suppressed by inhibiting NOX, and also by pharmacological inhibition of the TRPM2 channel or genetic depletion of the TRPM2 expression. These results support critical roles of ROS generation and subsequent TRPM2 channel activation in NPs-induced NLRP3 inflammasome activation and IL-1β generation (Yao et al., 2016). Our recent study shows that zinc oxide NPs (ZnO-NPs) induced brain pericyte cell death, which was prevented by siRNA-mediated knockdown of the TRPM2 expression in cultured pericytes and in mice by TRPM2-KO (Jiang et al., 2017). ZnO-NPs induced pericyte cell death was also suppressed by inhibiting nitric oxide synthase and scavenging peroxynitrite. Moreover, our study provides evidence to show that ZnO-NPs-induced TRPM2 protein nitration acts as a molecular inducer of autophagy that mediates pericyte cell death (Jiang et al., 2017). Collectively, accumulating evidence shows important roles of oxidative stress and TRPM2 channel in NPs-induced cellular effects.

## Concluding Remarks

It is clear from the above discussion that epidemiological studies support association of PM2.5 exposure with increased risk to AD and age-related brain damage. Experimental studies consistently support causative relationships of PM2.5 exposure to AD and age-related pathologies and, in addition, identify engagement of multiple pathological factors such as oxidative stress and multiple pathways. Nonetheless, it is noticeable that the current understanding is largely piecemeal and remains poor with respect to the underlying molecular and signaling mechanisms. Emerging evidence also suggests important roles of oxidative stress and the TRPM2 channel in mediating various NPs-induced cellular effects, prompting an attractive hypothesis that oxidative stress and the TRPM2 channel play similar roles in mediating PM2.5 exposure-induced AD predisposition and age-related brain damage. Further investigations are required to support or refute this hypothesis. Our hypothesis, if proves true, raises an interesting perspective on targeting the TRPM2 channel as a preventative and therapeutic strategy to limit the risk of PM2.5 exposure to AD and age-related brain damage in humans.

## Author Contributions

L-HJ proposed and elaborated the concept and drafted the manuscript. LuW and YY elaborated the concept and revised the manuscript. All authors participated in the discussion, and revised and approved the manuscript.

## Conflict of Interest

The authors declare that the research was conducted in the absence of any commercial or financial relationships that could be construed as a potential conflict of interest.
